# Unveiling a Rare Oropharyngeal Mass: A Case of Lymphangiomatous Polyp

**DOI:** 10.7759/cureus.107390

**Published:** 2026-04-20

**Authors:** Syed H Abbas, Akhila Aravind

**Affiliations:** 1 Pathology, University of Mississippi Medical Center, Jackson, USA

**Keywords:** anterior tonsillar fossa mass, lymphangiomatous polyp, oropharynx, polyp, tonsil

## Abstract

Lymphangiomatous polyps are rare benign lesions typically arising from nasal and laryngeal sites; however, oropharyngeal involvement is uncommon. We report the case of a 48-year-old male who presented with a stroke-like episode and was incidentally found to have a 3.0 × 1.4 × 2.4 cm pedunculated mass in the oropharynx. Flexible laryngoscopy revealed a smooth, pink, fleshy lesion near the anterior tonsillar fossa. Surgical excision was performed, and histopathology confirmed a lymphangiomatous polyp, supported by immunohistochemical stains (CD34 and ERG highlighting vascular structures). Although these lesions are benign, their potential for growth and airway compromise underscores the need for prompt recognition and definitive surgical excision.

## Introduction

Benign tumors of the palatine tonsil are exceedingly uncommon compared to malignant lesions, which constitute the majority of tonsillar masses. Among benign lesions, squamous papillomas are most frequently encountered, whereas lymphangiomatous polyps represent a rare subset of vascular and lymphatic malformations [1]. These lesions are characterized by a disorganized proliferation of lymphatic and vascular channels within a fibrous or adipose stroma. Historically, they have been described under several names, including “fibrovascular polyp,” “polypoid lymphangioma,” and “angiofibroma,” leading to confusion in the literature and potential underreporting [2,3]. Lymphangiomatous polyps usually arise in the nasal cavity, nasopharynx, or larynx; true oropharyngeal origin, particularly from the palatine tonsil or tonsillar pillars, is rare [4]. They typically present in the first two decades of life, though cases in middle-aged adults have also been reported [5,6]. These lesions may clinically mimic other benign and malignant tonsillar or oropharyngeal masses, including squamous papilloma, fibroepithelial polyp, vascular malformations, lymphoma, and squamous cell carcinoma. Clinically, these lesions may present as asymptomatic masses or produce nonspecific symptoms such as foreign body sensation, dysphagia, or intermittent bleeding. Due to their polypoid appearance and occasional growth, they may mimic malignant neoplasms.

We present a case of an incidentally discovered lymphangiomatous polyp of the oropharynx in a middle-aged male undergoing evaluation for acute neurological symptoms. This case highlights the importance of recognizing this rare benign entity and correlating clinical, radiologic, and histopathologic findings to guide appropriate management.

## Case presentation

A 48-year-old male presented as a stroke alert due to acute left-sided weakness, numbness, and dizziness. On arrival, the National Institutes of Health Stroke Scale (NIHSS) score was 4 for left arm drift, left leg drift, mild facial droop, and left-sided sensory deficit. Non-contrast CT of the head revealed no acute findings; CT angiography (CTA) of the head and neck showed no large vessel occlusion. He was outside the thrombolytic window, and no thrombectomy target was identified. He was admitted to the vascular neurology service for further evaluation. During the stroke workup, a right oropharyngeal mass was incidentally noted on imaging. CTA of the neck revealed a 2.2 × 1.6 × 3.2 cm mass within the posterior oropharynx extending toward the superior hypopharynx without significant enhancement on the arterial phase.

**Figure 1:**
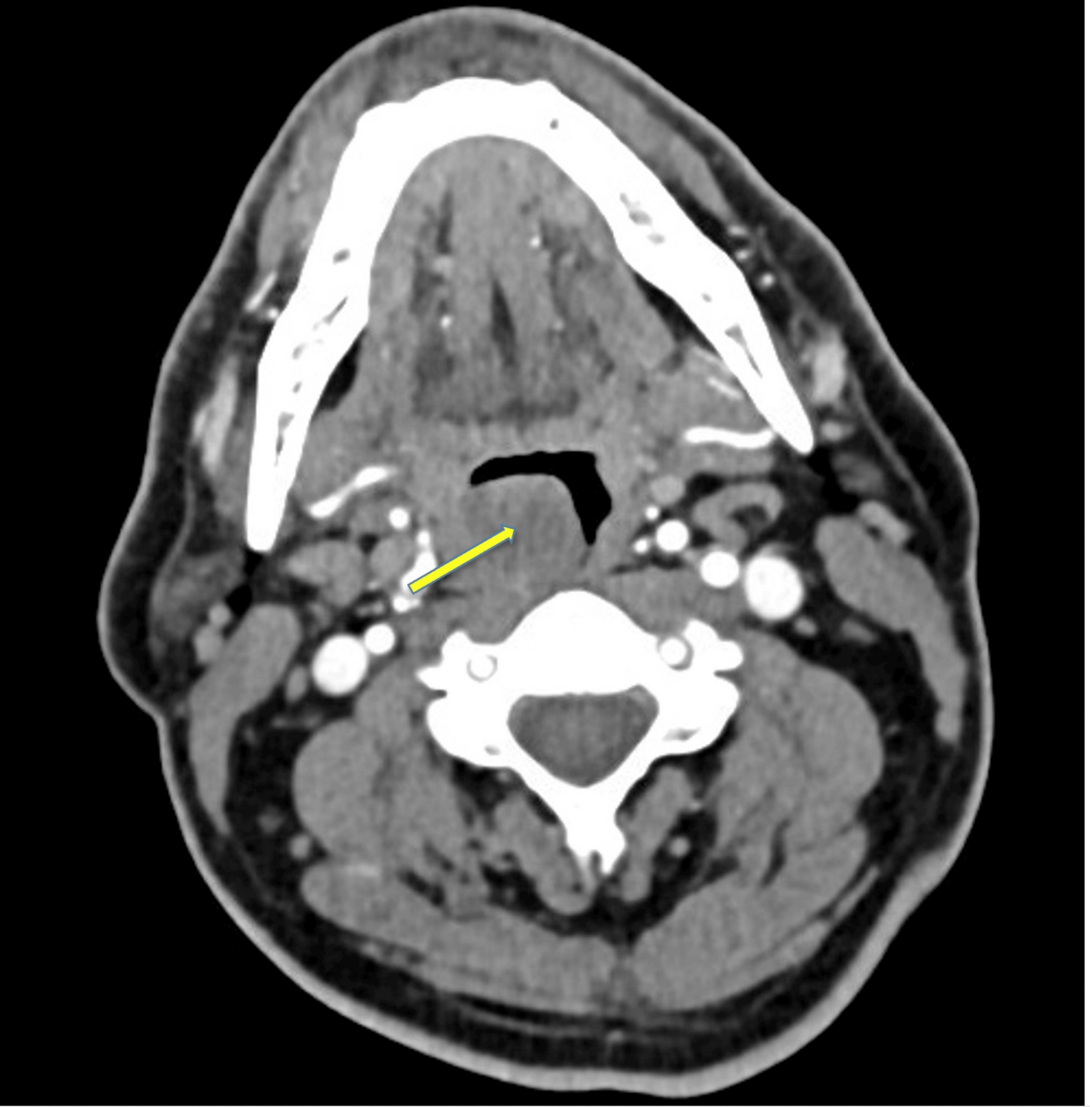
CT of the neck showing the mass

A subsequent contrast-enhanced CT soft tissue neck scan demonstrated a 3.0 × 1.4 × 2.4 cm pedunculated lesion prolapsing into the right lateral oral cavity and indenting the lateral aspect of the tongue (Figure 1). On physical examination, the patient was afebrile and hemodynamically stable.

**Figure 2:**
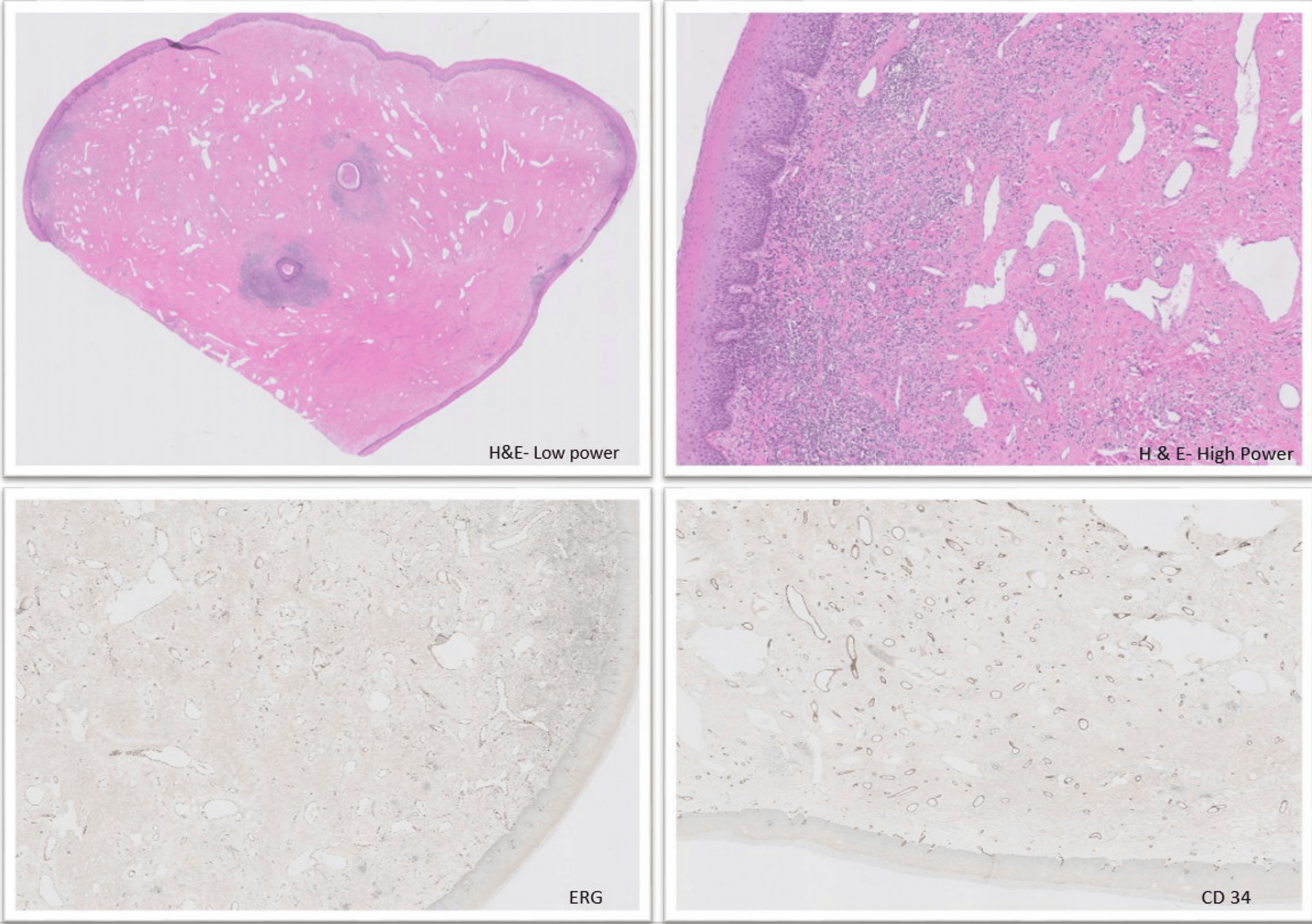
H&E sections of the polyp demonstrating characteristic histologic features, along with immunohistochemical staining for ERG and CD34 highlighting vascular structures H&E, hematoxylin and eosin

Neurological deficits improved to an NIHSS score of 1 within 24 hours. Oropharyngeal inspection revealed a smooth, pink, fleshy mass lateral to the right side of the oral tongue. Flexible fiber-optic laryngoscopy identified a pedunculated, non-ulcerated lesion originating from the superior pole of the right tonsil, with no extension to adjacent structures. The patient denied dysphagia, odynophagia, or respiratory difficulty but reported occasional bleeding when accidentally biting the lesion. Given the risk of recurrent bleeding while on dual antiplatelet therapy (aspirin and clopidogrel), otolaryngology was consulted. The patient underwent transoral excision of the mass under general anesthesia. Intraoperatively, a soft, pink pedunculated mass was seen attached by a narrow stalk to the superior pole of the right tonsil. The stalk was clamped with an Allis forceps and divided with electrocautery; hemostasis was achieved with suction cautery. No injury to the lips, teeth, or oral mucosa occurred. The estimated blood loss was minimal. The gross specimen measured 3.0 × 1.4 × 2.4 cm, with a smooth and polypoid appearance. Histopathologic examination revealed a proliferation of dilated lymphatic and vascular channels lined by benign endothelium within fibrous and adipose stroma, covered by keratinizing squamous epithelium without dysplasia. Lymphoid aggregates were present in the stroma. Immunohistochemical stains for CD34 and ERG highlighted endothelial cells of vascular channels (Figure 2), while CD3 and CD20 highlighted mixed T- and B-lymphocyte populations within the lesion. Surgical margins were free of lesion. The patient’s postoperative recovery was uneventful. He tolerated oral intake on postoperative day 1 and was discharged back to the correctional facility on his dual antiplatelet regimen. As the patient returned to the correctional facility and was not available for further outpatient follow-up, long-term follow-up data were unavailable.

## Discussion

Lymphangiomatous polyps represent a rare entity, accounting for approximately 2% of all tonsillar tumors [1]. The pathogenesis remains uncertain; hypotheses include developmental malformation of lymphatic drainage, localized lymphatic obstruction secondary to inflammation, and congenital sequestration of lymphatic tissue [7,8]. The lesion’s hamartomatous character, representing a benign disorganized proliferation of native tissue elements, is supported by its admixture of vascular, lymphatic, and connective tissue components. Clinically, these polyps present as painless, slow-growing masses that may cause foreign-body sensation, dysphagia, snoring, or mild bleeding. In rare instances, large lesions may cause airway obstruction or sleep-disordered breathing [9]. The differential diagnosis includes squamous papilloma, fibroepithelial polyp, vascular malformations, lymphoma, and squamous cell carcinoma. Histologically, lymphangiomatous polyps show dilated lymphatic channels lined by endothelial cells, embedded in fibrous and adipose stroma with lymphoid aggregates. The overlying squamous epithelium is typically intact and non-dysplastic. Immunohistochemical stains such as CD31, CD34, ERG, and D2-40 assist in confirming lymphatic differentiation [10]. A review of published literature reveals fewer than 50 reported cases worldwide. Kardon et al. analyzed 26 cases, with patients ranging from 3 to 63 years old (mean 25 years) and a slight male predominance [1]. Most presented with unilateral tonsillar enlargement, and all were treated successfully with local excision. Published reports have generally described no recurrence after complete excision. Our case adds to this limited body of evidence by describing an incidentally discovered lesion in an adult male undergoing evaluation for unrelated neurological symptoms. This underscores the importance of comprehensive head and neck evaluation when incidental findings arise on imaging. Furthermore, the benign histopathology and absence of recurrence reaffirm that simple excision is curative, avoiding the morbidity of unnecessary tonsillectomy or radical surgery.

## Conclusions

Lymphangiomatous polyps of the oropharynx are rare, benign, non-neoplastic lesions characterized by a disorganized proliferation of lymphatic channels that can clinically and radiographically mimic more aggressive pathologies. Their recognition requires awareness among clinicians and radiologists to prevent overdiagnosis and overtreatment. Histopathologic confirmation remains the gold standard for diagnosis. Complete surgical excision provides excellent long-term outcomes, with negligible risk of recurrence. This case reinforces the value of correlating radiologic, endoscopic, and histologic findings to achieve an accurate diagnosis. Early identification and management are particularly crucial in patients on antiplatelet therapy, where even minor lesions may pose bleeding risks.
